# Insensibility during Surgical Operations, by the Inhalation of an Ethereal Vaper

**Published:** 1847-01

**Authors:** 


### ECLECTIC DEPARTMENT,

### AND SPIRIT OF THE MEDICAL PERIODICAL PRESS.

---

*Insensibility during Surgical Operations, by the Inhalation of an Ethereal Vapor.*—In our last number we promised to furnish our readers with some reports of cases in which surgical operations have been performed apparently without pain during a state of temporary insensibility produced by the inhalation of an Ethereal Vapor. We copy from the Boston Medical and Surgical Journal several articles on this subject, reserving farther remarks of our own for the editorial department.—*Ed. Buffalo Med. Jour.*

*Communication of* John C. Warren, M. D., *Prof. of Surgery in Mass. Med. College.*—Application has been made to me by R. H. Eddy, Esq., in a letter dated Nov. 30th, in behalf of Dr. W. T. G. Morton, to furnish an account of the operations witnessed and performed by me, wherein his new discovery for preventing pain was employed. Dr. M. has also proposed to me to give him the names of such hospitals as I know of in this country, in order that he may present them with the use of his discovery. These applications, and the hope of being useful to my professional brethren, especially those concerned in the hospitals which may have the benefit of Dr. M's. proposal, have induced me to draw up the following statement, and to request it may be made public through your Journal.

The discovery of a mode of preventing pain in surgical operations, has been an object of strong desire among surgeons from an early period. In my surgical lectures I have almost annually alluded to it, and stated the means which I have usually adopted for the attainment of this object. I have also freely declared, that notwithstanding the use of very large doses of narcotic substances, this desideratum has never been satisfactorily obtained. The successful use of any article of the materia medica for this purpose, would therefore be hailed by me as an important alleviation to human suffering. I have in consequence readily admitted the trial of plans calculated to accomplish this object, whenever they were free from danger.

About five weeks since, Dr. Morton, dentist of this city, informed me that he had invented an apparatus for the inhalation of a vapor, the effect of which was to produce a state of total insensibility to pain, and that he had employed it successfully in a sufficient number of cases in his practice to justify him in a belief of its efficacy. He wished for an opportunity to test its power in surgical operations, and I agreed to give him such an opportunity as soon as practicable.

Being at that time in attendance as Surgeon of the Massachusetts General Hospital, a patient presented himself in that valuable institution a few days after my conversation with Dr. Morton, who required an operation for a tumor of the neck, and agreeably to my promise I requested the attendance of Dr. M,

On October 17th, the patient being prepared for the operation, the apparatus was applied to his mouth by Dr. Morton for about three minutes, at the end of which time he sank into a state of insensibility. I immediately made an incision about three inches long through the skin of the neck, and began a dissection among important nerves and blood-vessels without any expression of pain on the part of the patient. Soon after he began to speak incoherently, and appeared to be in an agitated state during the remainder of the operation. Being asked immediately afterwards whether he had suffered much, he said that he felt as if his neck had been scratched; but subsequently, when inquired of by me, his statement was, that he did not experience pain at the time, although aware that the operation was proceeding.

The effect of the gaseous inhalation in neutralizing the sentient faculty was made perfectly distinct to my mind by this experiment, although the patient during a part of its prosecution exhibited appearances indicative of suffering. Dr. Morton had apprised me, that the influence of its application would last but a few minutes after its intermission; and as the operation was necessarily protracted, I was not disappointed that its success was only partial.

On the following day, October 18th, an operation was done by Dr. Hayward, on a tumor of the arm, in a female patient at the Hospital. The respiration of the gas was in this case continued during the whole of the operation. There was no exhibition of pain, excepting some occasional groans during its last stage, which she subsequently stated to have arisen from a disagreeable dream. Noticing the pulse in this patient before and after the operation, I found it to have risen from 80 to 120.

Two or three days after these occurrences, on meeting with Dr. Charles T. Jackson, distinguished for his philosophical spirit of inquiry, as well as for his geological and chemical science, this gentleman informed me that he first suggested to Dr. Morton the inspiration of ether, as a means of preventing the pain of operations on the teeth. He did not claim the invention of the apparatus, nor its practical application; for these we are indebted to Dr. Morton.

The success of this process in the prevention of pain for a certain period being quite established, I at once conceived it to be my duty to introduce the apparatus into the practice of the Hospital, but was immediately arrested by learning that the proprietor intended to obtain an exclusive patent for its use. It now became a question, whether, in accordance with that elevated principle long since introduced into the medical profession, which forbids its members to conceal any useful discovery, we could continue to encourage an application we were not allowed to use ourselves, and of the components of which we were ignorant. On discussing the matter with Dr. Hayward, my colleague in the Hospital, we came to the conclusion, that we were not justified in encouraging the further use of this new invention, until we were better satisfied on these points. Dr. Hayward thereupon had a conversation with Dr. Morton, in consequence of which Dr. M. addressed to me a letter. In this he declared his willingness to make known to us the article employed, and to supply assistance to administer the inhalation whenever called upon. These stipulations he has complied with.

This being done, we thought ourselves justified in inviting Dr. Morton to continue his experiments at the Hospital, and elsewhere; and he directly after, Nov. 7th, attended at a painful and protracted operation performed by me, of the excision of a portion of the lower jaw, in which the patient's sufferings were greatly mitigated. On the same day an amputation of the thigh of a young woman was performed at the Hospital by Dr. Hayward. In this case the respiration of the ethereal vapor appeared to be entirely successful in preventing the pain of the operation; the patient stating afterwards, that she did not know that anything had been done to her.

On Nov. 12th, an operation for the removal of a tumor from the arm of a young woman was performed by Dr. J. Mason Warren. The vapor was administered for three minutes, when the patient became unconscious; the operator then proceeded, the inspiration being continued. Standing myself on one side of the patient, while the operator was on the other, so entirely tranquil was she, that I was not aware the operation had begun, until it was nearly completed.

On Nov. 21st, an operation was performed by Dr. J. Mason Warren on a gentleman for the removal of a tumor, which covered nearly the half of the front of the right thigh. The patient lying upon a bed, the vapor was administered by Dr. Morton in the presence of Drs. Charles T. Jackson, Reynolds, J. V. C. Smith, Flagg, Gould, Shurtleff, Lawrence, Parsons, Briggs, and others. After he had breathed the vapor for three minutes his head fell, and he ceased to respire it, but presently awaking, the inhalation was renewed till he again appeared insensible. The operation was then commenced. At the first stroke of the knife he clapped his hand on the wound, but I immediately seized and held it during the remainder of the operation, though not without some difficulty in consequence of his struggles. The operation was completed in two or three minutes, and the patient remained quietly on his back with his eyes closed. On examination the pupils were found to be dilated; the pulse was not materially affected. After he had lain about two minutes I roused him by the inquiry, "how do you do to-day?" to which he replied, "very well, I thank you." I then asked what he had been doing? He said he believed he had been dreaming; he dreamed that he was at home, and making some examination into his business. "Do you feel any pain?" "No." "How is that tumor of yours?" The patient raised himself in bed, looked at his thigh for a moment, and said, "It is gone, and I am glad of it." I then inquired if he had felt any pain during the operation, to which he replied in the negative. He soon recovered his natural state, experienced no inconvenience from the inhalation, was remarkably free from pain, and in three days went home into the country.

In all these cases there was a decided mitigation of pain; in most of them the patients on the day after the operation, and at other times, stated, that they had not been conscious of pain. All those who attended were, I think, satisfied of the efficacy of the application in preventing, or, at least, greatly diminishing the suffering usual in such cases. The phenomena presented in these operations afford grounds for many interesting reflections, but it being my principal intention at this time to give a simple statement of facts, I shall not pursue the subject further, but close with two or three remarks.

1st. The breathing of the ethereal vapor appears to operate directly on the cerebral system, and the consequent insensibility is proportionate to the degree of cerebral affection.

2d. Muscular power was for the time suspended in some cases, in others its loss was partial, and in one instance was scarcely sensible. The great relaxation of muscular action produced by a full dose of the application, leads to the hope that it may be employed with advantage in cases of spasmodic affection, both by the surgeon and by the physician.

3d. The action of the heart is remarkably accelerated in some cases, but not in all.

4th. The respiration is sometimes stertorous, like that of apoplexy.

All these changes soon pass off without leaving any distinct traces behind them, and the ordinary state of the functions returns. This has been the course of things in the cases I have witnessed, but I think it quite probable, that so powerful an agent may sometimes produce other and even alarming effects. I therefore would recommend, that it should never be employed except under the inspection of a judicious and competent person.

Let me conclude by congratulating my professional brethren on the acquisition of a mode of mitigating human suffering, which may become a valuable agent in the hands of careful and well-instructed practitioners, even if it shonld not prove of such general application as the imagination of sanguine persons would lead them to anticipate.

*Boston, Dec. 2d,* 1846.

*Communication of* A. L. Peirson, M. D., *of Salem, Mass.*
*To the Editor of the Boston Med. and Surg. Journal,*

Dear Sir,—The two following cases, occurring in my practice the past week, are of interest as supporting the claims to confidence of Dr. Morton's anodyne compound.

Case I.—Nov. 19th. An Irish girl, under 20 years of age, in attempting to step into the cars at Hamilton, while they were in motion, fell, with her arm upon the track, and had a compound, comminuted fracture at the elbow, from the wheel of the car. At about 9 in the evening, I amputated in the middle of the humerus. The operation lasted a little longer than if done by daylight, although it was a flap operation and quickly executed. Three vessels were tied. Dr. Fisk, dentist of this city, accompanied me, and caused the patient to inhale the vapor of the compound, about three minutes before the operation commenced. By this time she appeared to have yielded entirely to its influence, and became pale, silent and perfectly passive and manageable, whereas she had before exhibited evidence of great physical suffering and uncontrollable grief. Before the arteries were all tied she appeared to be returning to consciousness, when, on offering the apparatus to her mouth, she seized it with avidity, respired rapidly, and soon seemed to relapse into the unconscious state. It was thus renewed four or five times before she was placed in bed. Her own statement is that she suffered no pain during the operation, that she was asleep, and when she awoke she breathed again what was offered to her and fell asleep again—that she remembers to have done this three times. She says she did not know what we were doing to her, but in her sleep she thought she had got a reaping-hook in her arm, and that she heard the noise of sawing wood. She says she was not sensible of any thing till she was laid in bed, when she became quite talkative, and evidently somewhat excited. She slept some hours during the night. On dressing the stump on the third day, she made a violent outcry at the slighest pain. I was convinced that her statements with regard to her freedom from pain during the operation, were to be believed.

II.—Nov. 21st. An intelligent tanner, about 30 years old, had, with a fracture of both bones in the middle of the left leg, his ancle crushed by the cars engaged in building the Salem and Methuen Railroad. I amputated the leg just below the knee. The patient respired the vapor under Dr. Fisk's directions. He says he was not conscious of feeling any pain—and after the operation was finished and the ligatures applied, his consciousness returned, and with great apparent sincerity, he asked *if his limb was taken off*. He says, though he felt no pain, he was conscious of the presence of those around him, and he was obedient to the directions given him. The operation was performed at about 3, P. M., and the stump was dressed at about 9, when, he says, the pain of a few sutures far exceeded that of the operation.

In both these cases the pulse became somewhat accelerated after the operation, the countenance assumed a vacant expression, although in the first case there was working of the brows, and the pupils became dilated. They both appear to be doing well, and exhibit no symptoms worthy of note.

Respectfully yours,

*Salem, Nov. 24th,* 1846. A. L. PEIRSON.

*Postscript.—November 25th,* 1846.

Yesterday, I made further trial of the ethereal vapor, upon a middle-aged female, from whom I removed an adipose tumor, by an incision four inches long over the clavicle and scapula. She was an unimpressible subject, and was less perfectly under the influence of the vapor than the others, but she was entirely bewildered and not able to realize the nature of what we were doing to her. She was much more quiet than patients usually are, although the dissection was somewhat protracted, by the dipping down of the tumor into the supra spinal fossa of the clavicle, and confinement by fascia. She says she felt no pain, and did not evince any perception of the needle in dressing the wound—a sensation which usually calls forth complaint, as it is commonly unexpected.

It needs, no doubt, still further careful observation of its effects, to establish medical confidence in the new remedy, a confidence which must be of slow growth. From the results I have seen at the Massachusetts General Hospital, and in my own practice, I am led to expect the following advantages from its exhibition:

1st. Uniformity of its effects, unlike any mode of intoxication by stimulants in the stomach, or respiration of nitrous-oxide gas. My three patients were as unlike in age, temperament, and habits, as could well be imagined, yet all exhibited the same appearance of passive endurance.

2d. There was no instinctive or voluntary resistance, which is so embarrassing to an operator. This, next to its power of preventing the perception of pain, is the greatest merit claimed for it.

3d. The securing of the patient from the severity of the great shock which a capital operation inflicts on the sufferer. It was quite noticeable, in all the patients I have seen, that there was none of that extreme depression which sometimes follows a painful impression on the nervous system.

4th. Its effects pass off rapidly, and, as far as I know, no bad results will follow.

5th. It can be repeated several times during the operation, except the mouth or jaws are the parts to be operated on. The repetition of the dose is always sought by the patients with avidity.

6th. The last and most important of its effects is, that it either wholly annuls pain, or destroys the consciousness of it, so that it is not remembered; and thus the sentiment of fear is wholly obliterated. The patient appears to have been dreaming, and in the second case said that "he was in a distinct existence" (i. e., distinct from his former experience,) thus illustrating the theory of double consciousness.

These are recommendations enough to ensure it a fair trial among the humane and enlightened members of our profession, and for their decision we must wait, and by it be governed in its future use. Dr. Morton and Dr. Jackson, at least, are entitled to the hearty thanks of the profession for their discovery, and in the liberal manner in which they have offered it to all the subjects of surgical operations, both in and out of the Hospital. If some hunter up of obsolete theories should prove that such a thing had before been thought of, or tried, still these gentlemen are entitled to the credit of having made it, for the first time, perfectly available to the suffering, and submitted it to the test of those competent to decide on its merits, without being content to rest its pretensions on non-professional credulity or popular notoriety. A. L. Peirson.

*Salem, November 26th*, 1846.

---

*Communication of* H. J. Bigelow, M. D., *one of the Surgeons of Mass. General Hospital.* — We omit part of Dr. Bigelow's article, limiting ourselves to his account of the experiments which he has observed, and his remarks thereupon.

It remains briefly to describe the process of inhalation by the new method, and to state some of its effects. A small two-necked glass globe contains the prepared vapor, together with sponges to enlarge the evaporating surface. One aperture admits the air to the interior of the globe, whence, charged with vapor, it is drawn through the second into the lungs. The inspired air thus passes through the bottle, but the expiration is diverted by a valve in the mouth-piece, and escaping into the apartment is thus prevented from vitiating the medicated vapor. A few of the operations in dentistry, in which the preparation has as yet been chiefly applied, have come under my observations. The remarks of the patients will convey an idea of their sensations.

A boy of 16, of medium stature and strength, was seated in the chair. The first few inhalations occasioned a quick cough, which afterwards subsided; at the end of eight minutes the head fell back, and the arms dropped, but owing to some resistance in opening the mouth, the tooth could not be reached before he awoke. He again inhaled for two minutes, and slept three minutes, during which time the tooth, an inferior molar, was extracted. At the moment of extraction, the features assumed an xepression of pain, and the hand was raised. Upon coming to himself he said he had had a "first-rate dream—very quiet," he said, "and had dreamed of Napoleon—had not the slighest consciousness of pain—the time had seemed long;" and he left the chair, feeling no uneasiness of any kind, and evidently in a high state of admiration. The pupils were dilated during the state of unconsciousness, and the pulse rose from 130 to 142.

A girl of 16 immediately occupied the chair. After coughing a little, she inhaled during three minutes, and fell asleep, when a molar tooth was extracted, after which she continued to slumber tranquilly during three minutes more. At the moment when force was applied she flinched and frowned, raising her hand to her mouth, but said she had been dreaming a pleasant dream and knew nothing of the operation.

A stout boy of 12, at the first inspiration coughed considerably, and required a good deal of encouragement to induce him to go on. At the end of three minutes from the first fair inhalation, the muscles were relaxed and the pupil dilated. During the attempt to force open the mouth he recovered his consciousness, and again inhaled during two minutes, and in the ensuing one minute two teeth were extracted, the patient seeming somewhat conscious, but upon actually awaking he declared "it was the best fun he ever saw," avowed his intention to come there again, and insisted upon having another tooth extracted upon the spot. A splinter which had been left, afforded an opportunity of complying with his wish, but the pain proved to be considerable. Pulse at first 110, during sleep 96, afterwards 144; pupils dilated.

The next patient was a healthy-looking, middle-aged woman, who inhaled the vapor for four minutes; in the course of the next two minutes a back tooth was extracted, and the patient continued smiling in her sleep for three minutes more. Pulse 120, not affected at the moment of the operation, but smaller during sleep. Upon coming to herself, she exclaimed that "it was beautiful—she dreamed of being at home—it seemed as if she had been gone a month." These cases which occurred successively in about an hour, at the room of Dr. Morton, are fair examples of the average results produced by the inhalation of the vapor, and wlll convey an idea of the feelings and expressions of many of the patients subjected to the process. Dr. Morton states that in upwards of two hundred patients, similar effects have been produced. The inhalation, after the first irritation has subsided, is easy, and produces a complete unconsciousness at the expiration of a period varying from two to five or six, sometimes eight minutes; its duration varying from two to five minutes; during which the patient is completely insensible to the ordinary test of pain. The pupils in the cases I have observed have been generally dilated; but with allowance for excitement and other disturbing influences, the pulse is not affected, at least in frequency; the patient remains in a calm and tranquil slumber, and wakes with a pleasurable feeling. The manifestation of consciousness or resistance I at first attributed to the reflex function, but I have since had cause to modify this view.

It is natural to inquire whether no accidents have attended the employment of a method so wide in its application, and so striking in its results. I have been unable to learn that any serious consequences have ensued. One or two robust patients have failed to be affected. I may mention as an early and unsuccessful case, its administration in an operation performed by Dr. Hayward, where an elderly woman was made to inhale the vapor for at least half an hour without effect. Though I was unable at the time to detect any imperfection in the process, I am inclined to believe that such existed. One woman became much excited, and required to be confined to the chair. As this occurred to the same patient twice, and in no other case as far as I have been able to learn, it was evidently owing to a peculiar susceptibility. Very young subjects are affected with nausea and vomiting, and for this roason Dr. M. has refused to administer it to children. Finally, in a few cases, the patient has continued to sleep tranquilly for eight or ten minutes, and once, after a protracted inhalation, for the period of an hour.

The following case, which occurred a few days since, will illustrate the probable character of future accidents. A young man was made to inhale the vapor, while an operation of limited extent, but somewhat protracted duration, was performed by Dr. Dix upon the tissues near the eye. After a good deal of coughing, the patient succeeded in inhaling the vapor, and fell asleep at the end of about ten minutes. During the succeeding two minutes the first incision was made, and the patient awoke, but unconscious of pain. Desiring to be again inebriated, the tube was placed in his mouth, and retained there about twenty-five minutes, the patient being apparently half affected, but, as he subsequently stated, unconscious. Respiration was performed partly through the tube and partly with the mouth open. Thirty-five minutes had now elapsed, when I found the pulse suddenly diminishing in force, so much so, that I suggested the propriety of desisting. The pulse continued decreasing in force, and from 120 had fallen to 96. The respiration was very slow, the hands cold, and the patient insensible. Attention was now of course directed to the return of respiration and circulation. Cold affusions, as directed for poisoning with alcohol, were applied to the head, the ears were syringed, and ammonia presented to the nostrils and administered internally. For fifteen minutes the symptoms remained stationary, when it was proposed to use active exercise, as in a case of narcotism from opium. Being lifted to his feet, the patient soon made an effort to move his limbs, and the pulse became more full, but again decreased in the sitting posture, and it was only after being compelled to walk during half an hour that the patient was able to lift his head. Complete consciousness returned only at the expiration of an hour. In this case the blood was flowing from the head, and rendered additional loss of blood unnecessary. Indeed the probable hemorrhage was previously relied on as a salutary in its tendency.

Two recent cases serve to confirm, and one I think to decide, the great utility of this process. On Saturday, the 7th Nov., at the Mass. General Hospital, the right leg of a young girl was amputated above the knee, by Dr. Hayward, for disease of this joint. Being made to inhale the preparation, after protesting her inability to do from the pungency of the vapor, she became insensible in about five minutes. The last circumstance she was able to recall was the adjustment of the mouth-piece of the apparatus, after which she was unconscious until she heard some remark at the time of securing the vessels—one of the last steps of the operation. Of the incision she knew nothing, and was unable to say, upon my asking her, whether or not the limb had been removed. She refused to answer several questions during the operation, and was evidently completely insensible to pain or other external influences. This operation was followed by another, consisting of the removal of a part of the lower jaw, by Dr. Warren. The patient was insensible to the pain of the first incision, though she recovered her consciousness in the course of a few minutes.

The character of the lethargic state, which follows this inhalation, is peculiar. The patient loses his individuality and awakes after a certain period, either entirely unconscious of what has taken place, or retaining only a faint recollection of it. Severe pain is sometimes remembered as being of a dull character; sometimes the operation is supposed by the patient to be performed upon somebody else. Certain patients, whose teeth have been extracted, remember the application of the extracting instruments; yet none have been conscious of any real pain.

As before remarked, the phenomena of the lethargic state are not such as to lead the observer to infer this insensibity. Almost all patients under the dentist's hands scowl or frown; some raise the hand. The patient whose leg was amputated, uttered a cry when the sciatic nerve was divided. Many patients open the mouth, or raise themselves in the chair, upon being directed to do so. Others manifest the activity of certain intellectual faculties. An Irishman objected to the pain, that he had been promised an exemption from it. A young man taking his seat in the chair and inhaling a short time, rejected the globe, and taking from his pockets a pencil and card wrote and added figures. Dr. M. supposing him to be affected, asked if he would now submit to the operation, to which the young man willingly assented. A tooth was accordingly extracted, and the patient soon after recovered his senses. In none of these cases had the patients any knowledge of what had been done during their sleep.

I am, as yet, unable to generalize certain other symptoms to which I have directed attention. The pulse has been, as far as my observations extends, unaltered in frequency, though somewhat diminished in volume, but the excitement preceding an operation, has, in almost every instance, so accelerated the pulse that it has continued rapid for a length of time. The pupils are in a majority of cases dilated; yet they are in certain cases unaltered, as in the above case of amputation.*

The duration of the insensibility is another important element in the process. When the apparatus is withdrawn at the moment of unconsciousness, it continues, upon the average, two or three minutes, and the patient then recovers completely or incompletely, without subsequent ill effects. In this sudden cessation of the symptoms, this vapor in the air tubes differs in its effects from the narcotics or stimulants in the stomach, and, as far as the evidence of a few experiments of Dr. Morton goes, from the ethereal solution when breathed. Lassitude, headache and other symptoms lasted for several hours, when this agent was employed.

But if the respiration of the vapor be prolonged much beyond the first period, the symptoms are more permanent in their character. In one of the first cases, that of a young boy, the inhalation was continued during the greater part of ten minutes, and the subsequent narcotism and drowsiness lasted more than an hour. In a case alluded to before, the narcotism was complete during more than twenty minutes, the insensibility approached to coma.

Such cases resemble those before quoted from Christison and other authors, and show that the cessation of the inhalation, after it has been prolonged for a length of time, does not produce a corresponding cessation of the symptoms; while, if the inhalation is brief, the insensibility ceases in a short time. Recovery, in the latter case, is not improbably due to the complete and rapid elimination of the vapor from the lungs; the more gradual return of consciousness, in the former case, to the presence of a larger quantity of unexhaled particles. A fact mentioned by Christison bears upon this point. This author states that insensibility from the presence of a large quantity of alcohol in the stomach, often gives place to a complete and sudden return of consciousness, when the alcohol is removed by the stomach pump. It is probable that the vapor of the new preparation ceases early to act upon the system, from the facility with which it is exhaled.

The process is obviously adapted to operations which are brief in their duration, whatever be their severity. Of these, the two most striking are, perhaps, amputations and the extraction of teeth. In protracted dissections, the pain of the first incision alone is of sufficient importance to induce its use; and it may hereafter prove safe to administer it for a length of time, and to produce a narcotism of an hour's duration. It is not unlikely to be applicable in cases

* Since the above was written, I find this irregularity of symptoms mentioned in the case of poisoning by alcohol. Dr. Ogston, according to Christison, has in vain attempted to group together and to classify the states of respiration, pulse, and pupil.

requiring a suspension of muscular action; such as the reduction of dislocations or of strangulated hernia: and finally it may be employed in the alleviation of functional pain, of muscular spasm, as in cramp and colic, and as a sedative or narcotic.

The application of the process to the performance of surgical operations, is, it will be conceded, new. If it can be shown to have been occasionally resorted to before, it was only an ignorance of its universal application and immense practical utility that prevented such isolated facts from being generalized.

It is natural to inquire with whom this invention originated. Without entering into details, I learn that the patent bears the name of Dr. Charles T. Jackson, a distinguished chemist, and of Dr. Morton, a skilful dentist, of this city, as inventors—and has been issued to the latter gentleman as proprietor.

It has been considered desirable by the interested parties that the character of the agent employed by them, should not be at this time announced, but it may be stated that it has been made known to those gentlemen who have had occasion to avail themselves of it.

I will add, in conclusion, a few remarks upon the actual position of this invention as regards the public.

No one will deny that he who benefits the world should receive from it an equivalent. The only question is, of what nature shall the equivalent be? Shall it be voluntarily ceded by the world, or levied upon it? For various reasons, discoveries in high science have been usually rewarded indirectly by fame, honor, position, and occasionally, in other countries, by funds appropriated for the purpose. Discoveries in medical science, whose domain approaches so nearly to philanthropy, have been generally ranked with them; and many will assent with reluctance to the propriety of restricting by letters patent the use of an agent capable of mitigating human suffering. There are various reasons, however, which apologize for the arrangement which I understand to have been made with regard to the application of the new agent.

1st. It is capable of abuse, and can readily be applied to nefarious ends.

2d. Its action is not yet thoroughly understood, and its use should be restricted to responsible persons.

3d. One of its greatest fields is the mechanical art of dentistry, many of whose processes are by convention, secret, or protected by patent rights. It is especially with reference to this art, that the patent has been secured. We understand, already, that the proprietor has ceded its use to the Mass. General Hospital, and that his intentions are extremely liberal with regard to the medical profession generally, and that as soon as necessary arrangements can be made for publicity of the process, great facilities will be offered to those who are disposed to avail themselves of what now promises to be one of the most important discoveries of the age.

